# Alien Vascular Plants of Kazakhstan: The First National Checklist and Spatial Distribution Patterns

**DOI:** 10.3390/plants15192934

**Published:** 2026-09-25

**Authors:** Daniyar Alibekov, Serik Kubentayev, Marcin Nobis, Victor Chepinoga, Margarita Ishmuratova, Alexander N. Sennikov

**Affiliations:** 1 Astana Botanical Garden, Astana 010000, Kazakhstan; dansilvaforest@mail.ru; 2 Laboratory “NatureLab”, Higher School of Natural Sciences, Astana International University, Astana 010000, Kazakhstan; kubserik@mail.ru; 3 Institute of Botany, Faculty of Biology, Jagiellonian University, 30-387 Kraków, Poland; m.nobis@uj.edu.pl; 4 Geobotany Section, Institute of Earth System Sciences (IESW), Leibniz University Hannover, 30419 Hannover, Germany; chepinoga@geobotanik.uni-hannover.de; 5 Faculty of Biology and Geography, Karaganda National Research University Named After Academician E.A. Buketov, Karaganda 100028, Kazakhstan; margarita.ishmur@mail.ru; 6 Botanical Museum, Finnish Museum of Natural History, University of Helsinki, 00014 Helsinki, Finland

**Keywords:** archaeophytes, biological invasions, Central Asia, EICAT, naturalization, neophytes, species inventories

## Abstract

Biological invasions contribute to global biodiversity change, yet Kazakhstan’s alien vascular flora remains insufficiently documented. We provide the first comprehensive verified inventory of alien vascular plants in Kazakhstan, synthesizing literature, herbarium collections, field observations, and iNaturalist and Plantarium records. The alien flora comprises 263 taxa in 181 genera and 54 families: 141 taxa (53.6%) are naturalized, 120 are casual, and two have unresolved establishment status. Neophytes predominate (218 taxa: 104 naturalized, 112 casual, two unresolved), whereas archaeophytes comprise 45 taxa (37 naturalized, eight casual). Asteraceae is the most taxon-rich family (47 taxa), followed by Poaceae (30), Brassicaceae and Fabaceae (20 each), Amaranthaceae (15), and Lamiaceae (14), while annual taxa dominate the life-cycle spectrum. Mediterranean-origin taxa form the largest biogeographical group (39.9%), followed by taxa native to the Americas (22.8%). Most taxa are associated with human-modified habitats; 75 taxa (28.5%) also occur in natural ecosystems. Twenty taxa are invasive; under the Environmental Impact Classification for Alien Taxa (EICAT), 17 have Moderate and three Major impacts. Alien taxon richness is spatially heterogeneous, with the highest values in the Trans-Ili Alatau, Altai, and Western Tian-Shan. This study establishes a national baseline for monitoring, invasion-risk assessment, biodiversity management, and conservation in Kazakhstan and Central Asia.

## 1. Introduction

Invasive alien plants can substantially alter the structure and composition of plant communities, reduce native species diversity, and modify ecosystem processes [1,2]. Their impacts may include changes in biogeochemical cycles, trophic interactions, successional processes, and ultimately the biotic homogenization of ecosystems [3,4,5]. Biological invasions by alien plants can also generate considerable economic costs through impacts on agriculture, infrastructure, and natural-resource use [6], while some species pose risks to human health because of allergenic or toxic properties [7,8].

The introduction of plant species beyond their native ranges has accelerated with the expansion of international trade, urbanization, and transportation networks [9]. Alien plants may be introduced intentionally as ornamental, forage, industrial, medicinal, or food plants, or unintentionally as contaminants of agricultural commodities, seed material, soil, and transported goods [10,11]. Following introduction, some species persist only temporarily, whereas others establish self-sustaining populations and may subsequently spread beyond the sites of introduction [12]. The dynamics of this process can be difficult to detect because some alien species exhibit prolonged lag phases before undergoing rapid range expansion [13].

National and regional inventories of alien plants provide an essential basis for assessing biological invasions, documenting establishment and spread, and developing monitoring and management priorities [14,15]. At the global scale, the Global Naturalized Alien Flora (GloNAF) database has greatly improved knowledge of the distribution of naturalized alien plants across more than one thousand regions worldwide [16]. Nevertheless, Central Asia remains comparatively understudied with respect to alien plant diversity and invasion processes. A comprehensive inventory of the alien flora of Kyrgyzstan documented 151 alien species, of which 58 (38.4%) were naturalized [17], with additional taxonomic and distributional records subsequently published by Sennikov et al. [18]. In Uzbekistan, 252 alien species have been documented, including 31 casual, 177 naturalized non-invasive, and 44 invasive species [19]. In comparison, the alien floras of Tajikistan and Turkmenistan remain less comprehensively synthesized at the national level, although numerous floristic, herbarium, and taxonomic records document alien plant occurrences throughout these countries [20,21,22,23,24,25].

Despite a long history of botanical exploration, Kazakhstan has lacked a critically verified analytical synthesis of its alien vascular flora at the national scale. Available information has remained dispersed among regional floras, weed and quarantine inventories, floristic records, herbarium collections, and biodiversity databases. Earlier treatments have also differed in their interpretation of native and alien status, particularly for archaeophytes and other long-established taxa. Historical movements of crop seeds, forage plants, planting material, and other commodities during the Russian Empire and Soviet periods played an important role in the accumulation of alien plants across Central Asia [17], while ornamental species introduced for cultivation and urban greening have subsequently escaped and become established in parts of Kazakhstan [26,27,28]. Although numerous regional studies and individual floristic records are available [26,28,29,30,31,32,33], a unified national assessment integrating taxonomy, establishment status, residence time, ecological attributes, and spatial distribution has been lacking.

Kazakhstan represents an important region for invasion research owing to its large geographic extent, pronounced environmental heterogeneity, and long history of human land use. Agricultural development, large-scale cultivation of previously uncultivated lands, railway construction, expansion of transport infrastructure, mining, urbanization, and urban greening have substantially transformed many parts of the country and facilitated the introduction and spread of alien plant species [9,26,27,28]. Kazakhstan occupies the central part of Eurasia and is the ninth largest country in the world, covering an area of 2,724,900 km^2^. Its vast territory encompasses pronounced climatic, topographic, and ecological gradients and several major biogeographical transition zones, including the transition from the cold-temperate and temperate regions of northern Eurasia to the warmer arid and semi-arid Irano-Turanian region in the south [34,35]. Phytogeographically, Kazakhstan lies at the junction of the Irano-Turanian and Circumboreal floristic regions of the Holarctic Kingdom. Most of the country belongs to the Irano-Turanian region of the Tethyan (Ancient Mediterranean) subkingdom, whereas northern Kazakhstan forms part of the Circumboreal region of the Boreal subkingdom. Southeastern Kazakhstan, including the Tian-Shan and Dzungarian Alatau mountain systems, is assigned to the Central Asian Mountain Province of the Tethyan subkingdom [36].

Here, we provide the first comprehensive and critically verified national inventory of alien vascular plants of Kazakhstan. Specifically, we aimed to (1) compile and taxonomically verify the national checklist of alien vascular plant taxa; (2) assess their taxonomic composition, life-cycle structure, geographic origin, residence time, and establishment status; (3) characterize their cultivation history, weed and habitat associations, and environmental impacts; and (4) analyse patterns of alien plant distribution across the floristic and administrative regions of Kazakhstan. This synthesis provides a baseline for monitoring future changes in the alien flora and for comparative analyses of plant invasions in Central Asia.

## 2. Results

The present study recorded 263 alien vascular plant taxa in Kazakhstan, belonging to 181 genera (Table S1). Of these, 141 taxa were classified as naturalized and 120 as casual aliens, whereas the status of two taxa remained unresolved. Overall, naturalized taxa accounted for 53.6% of the country’s alien flora. The alien flora of Kazakhstan comprised 54 vascular plant families and showed a strongly uneven taxonomic structure. Asteraceae was the most taxon-rich family (47 taxa), followed by Poaceae (30), Brassicaceae and Fabaceae (20 each), Amaranthaceae (15), and Lamiaceae (14). All remaining families contained fewer than ten alien taxa, with Solanaceae being the most diverse among them (9 taxa) (Figure 1).

Among naturalized alien taxa, Asteraceae was the most taxon-rich family (32 taxa), followed by Poaceae (22), Brassicaceae and Fabaceae (11 each), Amaranthaceae (8), Caryophyllaceae and Lamiaceae (6 each), Malvaceae (5), Solanaceae (4), and Hydrocharitaceae and Violaceae (3 each). Among casual taxa, Asteraceae was again the most diverse family (15 taxa), followed by Brassicaceae and Fabaceae (9 each), Lamiaceae (8), Amaranthaceae and Poaceae (7 each), Solanaceae (5), and Boraginaceae, Cyperaceae, Euphorbiaceae, and Rosaceae (4 each). The two taxa of unresolved establishment status belonged to Boraginaceae and Poaceae (Figure 1).

The dataset comprises 1727 distribution records documenting the occurrence of these alien taxa across the 29 floristic regions and seven floristic subregions of Kazakhstan. For each taxon, standardized information is provided on taxonomy, nomenclature, establishment status, and regional distribution (Table S1). Most alien taxa in Kazakhstan were classified as neophytes (218 taxa), including 104 naturalized, 112 casual, and two taxa of unresolved status, whereas 45 taxa were classified as archaeophytes, comprising 37 naturalized and eight casual taxa. Naturalization success differed markedly between the two groups, with archaeophytes showing a substantially higher naturalization rate (82.2%) than neophytes (47.7%).

The taxonomic structure of the alien flora was characterized by high generic diversity and generally low taxon richness within genera. Most genera were represented by a single alien taxon (131 genera), followed by 33 genera with two taxa and nine genera with three taxa. Only a few genera contained more than three alien taxa: *Amaranthus* was the most taxon-rich genus with nine taxa, followed by *Vicia* with six, whereas *Centaurea*, *Cuscuta*, *Euphorbia*, *Lolium*, *Setaria*, and *Solanum* were represented by four taxa each. Among naturalized alien taxa, *Amaranthus* and *Vicia* were the most diverse genera, with four taxa each, followed by *Ambrosia*, *Centaurea*, *Lolium*, *Malva*, *Setaria*, *Viola*, and *Xanthium*, with three taxa each. Within the casual alien fraction, *Amaranthus* was the most taxon-rich genus with five taxa, followed by *Euphorbia* with three, whereas 14 genera were represented by two casual taxa each. Among the genera shown in Figure 2, *Ambrosia*, *Malva*, *Viola*, and *Xanthium* were represented exclusively by naturalized alien taxa (Figure 2).

When classified by life-cycle duration, annual taxa dominated the alien flora of Kazakhstan, comprising 153 taxa (58.2%). Perennial polycarpic taxa accounted for 100 taxa (38.0%), perennial monocarpic taxa for eight taxa (3.0%), and biennial taxa for two taxa (0.8%). Among annual taxa, 89 were naturalized, 63 were casual, and one had unresolved status. Among perennial polycarpic taxa, 47 were naturalized, 52 were casual, and one had unresolved status. Casual taxa predominated among perennial monocarpic taxa (five versus three naturalized taxa), whereas both biennial taxa were naturalized.

When classified by geographic origin, the alien flora of Kazakhstan was dominated by taxa of Mediterranean origin (105 taxa; 39.9%). Taxa native to the Americas represented the second largest group (60 taxa; 22.8%), followed by those of European (34; 12.9%) and Asian origin (29; 11.0%). Considerably smaller contributions were made by taxa originating from the Asia Minor–Caucasus–Iran region (12; 4.6%), taxa introduced through cultivation (11; 4.2%), and Afro-Asiatic taxa (9; 3.4%) (Table 1). Taxa of African, Eurasian, and Asia–Australasian origin were represented by a single taxon each. The highest proportion of naturalized taxa was recorded among taxa of Mediterranean origin (70.5%), whereas the corresponding values for taxa of American, European, and Asian origin were 43.3%, 41.2%, and 44.8%, respectively.

The vast majority of alien plant taxa (232 taxa; 88.2%) originated from temperate regions, whereas taxa of subtropical and tropical origin together accounted for only 31 taxa (11.8%). Naturalization success was considerably lower among taxa of subtropical and tropical origin (9 of 31 taxa; 29.0%) than among those originating from temperate regions (132 of 232 taxa; 56.9%).

A total of 113 alien plant taxa in Kazakhstan (43.0%) had a documented history of cultivation (Table 2). Because some taxa had more than one documented use, cultivation-use categories were treated as non-mutually exclusive. Ornamental plants constituted the largest group (57 taxa; 21.7% of all alien taxa), followed by edible (24; 9.1%), fodder (19; 7.2%), medicinal (9; 3.4%), and technical plants (7; 2.7%). The remaining 150 taxa (57.0%) had no documented history of cultivation. Naturalization success differed among the cultivation-use groups. Edible plants showed the lowest naturalization rate (25.0%), ornamental and medicinal plants showed intermediate values of 40.4% and 44.4%, respectively, whereas fodder plants had the highest rate (63.2%). Taxa with no documented history of cultivation showed a naturalization rate of 62.0%, and technical plants 57.1%.

The distribution of alien plants in Kazakhstan showed a strong association with human-modified habitats. Among the 263 recorded alien taxa, 75 taxa (28.5%) were documented in natural ecosystems, whereas the remaining 188 taxa (71.5%) were confined to human-modified habitats.

According to the IUCN Environmental Impact Classification for Alien Taxa (EICAT) [37], most alien taxa occurring in natural ecosystems of Kazakhstan were assigned to the Minor (MN) impact category (37 taxa). The Minimal Concern (MC) category comprised 18 taxa, the Moderate (MO) category 17 taxa, and three taxa were assigned to the Major (MR) category. Thus, taxa with minimal or minor environmental impacts predominated among aliens recorded in natural ecosystems, while only three taxa were classified as having major environmental impacts. Among the 20 alien taxa identified as invasive in Kazakhstan, 17 were assigned to the Moderate (MO) and three to the Major (MR) EICAT impact categories (Table 3). More than half of these invasive taxa belonged to Asteraceae (11 taxa; 55%), whereas Convolvulaceae was represented by two taxa and all remaining families by a single taxon each. At the genus level, *Ambrosia* was the most taxon-rich genus (three taxa), followed by *Cuscuta*, *Erigeron*, and *Xanthium*, with two taxa each. Most invasive taxa were of American origin (12 taxa), followed by those of European origin (4), whereas Mediterranean and Asian origins were each represented by two taxa. Neophytes clearly predominated among invasive taxa (19 taxa), while only *Phelipanche aegyptiaca* (Pers.) Pomel was classified as an archaeophyte. In terms of life cycle, annual taxa predominated (11 taxa), followed by perennial taxa (8) and a single biennial taxon.

Weed taxa accounted for 91 taxa (34.6% of all recorded alien taxa) in the alien flora of Kazakhstan, 40 of which were classified as noxious weeds. Alien weed taxa were further categorized according to the types of agricultural crops in which they occurred, including cereals, vegetables, ornamentals, rice, and fodder crops. Cereal crops supported the largest number of alien weed taxa (43 taxa; 47.3%), followed by vegetable crops (28 taxa; 30.8%). Ornamental crops and rice fields were each associated with nine weed taxa (9.9%), whereas fodder crops were associated with only two taxa (2.2%). Overall, 60 of the 91 recorded alien weed taxa (65.9%) were classified as naturalized. The proportion of naturalized taxa was particularly high among weeds associated with vegetable crops (78.6%) and cereal crops (74.4%). Eighty-seven taxa were recorded in ruderal habitats, highlighting the important role of anthropogenically disturbed environments in the establishment and spread of alien weeds.

The distribution of alien plant taxa across the floristic regions of Kazakhstan was markedly heterogeneous (Figure 3). The highest numbers of alien taxa were recorded in the Trans-Ili Alatau (154 taxa; 58.6% of the total alien flora), followed by the Altai (113; 43.0%), Western Tian-Shan (105; 39.9%), and Western Upland (103; 39.2%). In contrast, the lowest numbers of alien taxa were recorded in Southern Ustyurt and Buzachi (5 taxa each; 1.9%), Northern Ustyurt (6; 2.3%), and Bukeev and Kyzylkum (14 each; 5.3%).

The representation of alien plant taxa varied markedly among the administrative regions of Kazakhstan (Figure 4). Almaty Region had the highest representation of alien taxa, with 158 taxa (60.1% of the total alien flora), followed by Turkestan Region with 124 taxa (47.1%), Zhetysu Region with 110 taxa (41.8%), East Kazakhstan Region with 109 taxa (41.4%), and Zhambyl Region with 106 taxa (40.3%). In contrast, Mangystau Region had the lowest representation, with only 25 alien taxa (9.5%), whereas the remaining administrative regions showed intermediate levels of alien taxon richness.

## 3. Discussion

The present study provides the first comprehensive inventory of the alien vascular flora of Kazakhstan and establishes a contemporary baseline for future research on the country’s alien flora. Despite the extensive use of herbarium collections, published sources, and original field observations, the checklist should be regarded as a dynamic resource that will continue to evolve as new floristic data become available, herbarium collections are critically revised, and the status of individual taxa is reassessed. This is particularly relevant for casual alien species, for which the absence of long-term observations often prevents a reliable assessment of their ability to establish self-sustaining populations [4,6,14].

To date, Kazakhstan has remained one of the largest countries in Eurasia that lacked a modern national synthesis of its alien flora. According to the Global Naturalized Alien Flora (GloNAF) database, which compiles information on naturalized alien plants from more than one thousand regions worldwide, Central Asia remains among the least studied regions in terms of alien plant diversity [16]. During the last few years, comparable national checklists have been published for Kyrgyzstan [17] and Uzbekistan [19]. The present study complements these efforts by providing the first standardized dataset for Kazakhstan, the largest country in Central Asia.

The alien flora consists almost entirely of terrestrial plants. The only aquatic species recorded were *Pistia stratiotes* L., *Eriocaulon cinereum* R.Br., *Elodea canadensis* Rich. ex Michx., *Najas graminea* Delile and *Vallisneria spiralis* L., which is in agreement with the low number of aquatic alien plant species registered in arid regions [38].

Analysis of the generic composition revealed that most genera are represented by a single alien species, whereas only a few contain multiple alien taxa. This pattern suggests that the alien flora of Kazakhstan has been assembled primarily through numerous independent introduction events involving representatives of diverse taxonomic groups rather than through extensive diversification within particular genera. The genus *Amaranthus* is the most species-rich, which is consistent with observations from both Kyrgyzstan [17] and Uzbekistan [19], where it also represents one of the dominant alien genera. This similarity further highlights the common pathways and processes underlying the formation of alien flora across Central Asia.

According to our assessment, 141 taxa (53.6%) are naturalized, whereas 120 taxa remain classified as casual aliens. In terms of the proportion of naturalized species, Kazakhstan occupies an intermediate position among neighbouring countries. Kyrgyzstan currently hosts 58 naturalized species out of 151 alien taxa [17], whereas Uzbekistan has 221 naturalized alien species in total (177 naturalized non-invasive and 44 invasive) out of 252 alien species [19]. These differences may reflect variations in environmental conditions, land-use history, and the degree of floristic exploration. In particular, Uzbekistan has recently undergone a comprehensive revision of its flora within the framework of the Flora of Uzbekistan project, accompanied by the development of a modern biodiversity database and grid-based mapping of plant distributions [39,40], thereby facilitating more complete detection of alien species and more accurate assessments of their invasion status.

According to the most recent checklist of the vascular flora of Kazakhstan [41], the native flora of the country comprises 5883 vascular plant taxa. When combined with the 263 alien taxa documented in the present study, alien plants account for approximately 4.3% of the total vascular flora of Kazakhstan. Although knowledge of the country’s alien flora continues to expand, this relatively low proportion suggests a comparatively limited degree of floristic alienization. Relatively low proportions of alien taxa have also been reported from other arid and semi-arid regions, including Kyrgyzstan (approximately 4%) [17], Iran (3.7%) [42], and Oman (7.7%) [43].

In contrast, alien species constitute a substantially larger proportion of the flora in several well-studied European countries, exceeding 64% in Finland [44], reaching 37.8% in the Czech Republic [45], approximately 27% in Poland, and 13.8% in Ukraine [46]. These differences may reflect a combination of climatic conditions, land-use history, human population density, propagule pressure, introduction history, survey intensity, and methodological differences among national inventories. The relatively low contribution of alien species to the flora of Kazakhstan may partly reflect its arid climate, the predominance of extensive steppe and desert ecosystems, and comparatively low human population density. Climatic conditions, particularly water availability, are known to influence plant establishment and naturalization, and arid environments may therefore constrain the establishment of some alien taxa [39,47]. However, the relative influence of climatic and anthropogenic factors in shaping the alien flora of Kazakhstan requires further investigation.

The life-form spectrum of the alien flora of Kazakhstan is clearly dominated by annual species. A similar pattern has been documented in both Kyrgyzstan and Uzbekistan [17,19], indicating a broadly comparable life-history structure among the alien floras of Central Asia. The predominance of annual plants is characteristic of many arid regions worldwide and reflects their ability to complete their life cycle rapidly, exploit short periods of favourable environmental conditions, and successfully colonize disturbed habitats. The high proportion of annuals is also consistent with the predominance of ruderal and segetal species in the alien flora of Kazakhstan.

Our analysis of the geographic origin of alien taxa revealed that species of Mediterranean origin constitute the largest group, followed by taxa native to the Americas. A similar predominance of Mediterranean elements has previously been reported for the alien flora of Kyrgyzstan [17], probably reflecting long-standing historical connections between the Mediterranean Basin and Central Asia, as well as similarities in climatic conditions across parts of these regions. The substantial contribution of American species is consistent with global invasion patterns [47], as numerous taxa native to North and South America have expanded far beyond their native ranges through the development of agriculture, transportation networks, and international trade. European taxa represent the third largest group. However, determining the origin of some European species is complicated by the fact that northern Kazakhstan belongs to the Circumboreal Region of the Boreal Kingdom [36], where the natural ranges of several European taxa extend into the country. Consequently, the native or alien status of such species requires careful reassessment in future taxonomic and floristic revisions.

The spatial distribution of alien plants across Kazakhstan is markedly heterogeneous. The highest numbers of alien taxa were recorded in Almaty, Turkestan, Zhetysu, East Kazakhstan, and Zhambyl administrative regions. A similar pattern is evident at the level of floristic regions, where the greatest species richness was observed in Trans-Ili Alatau, Altai, Western Tian-Shan, and several adjacent floristic regions. These areas are among the most intensively investigated botanical regions of Kazakhstan and are also characterized by relatively high human population density, well-developed transportation infrastructure, intensive agricultural activity, and a long history of plant introductions [28,48]. Therefore, the observed spatial patterns are likely to reflect both genuine differences in the distributions of alien species and unequal sampling intensity among different parts of the country.

The high species richness of alien plants in the Altai region is consistent with the results of long-term floristic and taxonomic studies conducted in that territory [49,50,51,52,53,54,55,56,57], which have resulted in the recent discovery of several alien species newly recorded in Kazakhstan, including *Elymus canadensis* L. and *Setaria maximowiczii* Tzvelev & Prob. Furthermore, the extensive floristic knowledge accumulated in the adjacent Russian Altai [27,58] has substantially improved our understanding of species distributions in the transborder regions of eastern Kazakhstan. Similarly, the high concentration of alien species in south-eastern Kazakhstan agrees with recent regional studies, including the first regional Black Book of Kazakhstan [28], as well as the rapidly increasing number of verified observations available through the iNaturalist platform.

Despite the growing interest in biological invasions, studies addressing the impacts of alien plants on natural plant communities in Kazakhstan remain scarce [59,60,61,62]. Among the first comprehensive investigations of anthropogenic transformation of natural vegetation in Kazakhstan were the studies conducted by Gorchakovskii in the Kazakh Uplands [63]. He demonstrated that an increase in the proportion of synanthropic species, both in terms of their abundance and their contribution to the total flora, accompanies the degradation of natural plant communities. In particular, this relationship was documented during the degradation of relict black alder forests in Central Kazakhstan, leading to the proposal that the proportion of synanthropic species can serve as an indicator of the degree of anthropogenic transformation of vegetation [64]. The modern concept of biological invasions has substantially expanded these views. Whereas an increasing proportion of synanthropic species was previously regarded mainly as an indicator of anthropogenic disturbance, it is now widely recognized that such disturbances also increase the susceptibility of natural communities to the introduction, naturalization, and spread of alien species [65]. For example, wildfires facilitate the invasion of *Acer negundo* L. in the Ural River floodplain [61], demonstrating that the disruption of natural ecosystem processes substantially increases ecosystem susceptibility to alien plant establishment. Conversely, some invasive plant species are capable of altering fire regimes by either increasing or decreasing fire frequency and intensity, depending on their functional traits. This creates positive feedback between ecosystem disturbance and biological invasions: fire promotes the establishment of invasive plants, whereas invasive plants modify fire regimes in ways that facilitate their further spread [66].

Studies conducted in Belarus and Russia have shown that the invasion by *Acer negundo* is accompanied by a decline in both total and native plant species richness while simultaneously increasing the proportion of alien species within invaded communities. These changes have been attributed to alterations in community structure that may facilitate the establishment of additional alien plants in habitats already modified by *A. negundo* [67]. Although comparable studies combining vegetation analysis with bioinformatic approaches have not yet been conducted in Kazakhstan, similar processes may occur in the country’s floodplain ecosystems following the spread of this species. If confirmed, this would indicate the existence of a positive feedback mechanism whereby the establishment of one invasive species promotes further synanthropization of plant communities, reduces their biotic resistance, and creates more favourable conditions for the naturalization of additional alien species. This mechanism is consistent with the invasional meltdown hypothesis, according to which some alien species facilitate the establishment and subsequent spread of other alien species, thereby accelerating ecosystem transformation [68]. Moreover, to our knowledge, no studies in Kazakhstan have yet applied mass spectrometry imaging [69,70,71], chromosomal-scale genome assembly [72,73,74,75], or spatial distribution modelling [76,77,78] to alien or invasive plant species. These emerging approaches could substantially enhance our understanding of invasion mechanisms and support monitoring. For example, mass spectrometry imaging could reveal the tissue-specific localisation of allelochemicals in aggressive invaders such as *Acer negundo*; genomic tools could identify adaptive traits facilitating its spread; and spatial modelling could predict its future range expansion under climate change.

On the basis of our results and the available literature, floodplain and riparian ecosystems appear to be the most vulnerable natural habitats to alien plant invasions in Kazakhstan. Gorchakovskii and Ryabinina demonstrated that steppe communities were more resistant to livestock grazing than meadow vegetation during anthropogenic degradation [79]. In addition to their lower resistance to overgrazing, meadow ecosystems receive a continuous influx of propagules transported by watercourses, reducing the biotic resistance of these communities and increasing their susceptibility to invasion by alien species such as *Bidens frondosa* L., *Hordeum jubatum* L., and *Juncus tenuis* Willd. In contrast, *Centaurea diffusa* Lam. preferentially colonizes well-drained light soils, and its abundance increases under conditions of overgrazing and pasture degradation because trampling creates suitable microsites for seed germination, as the species is largely avoided by livestock and is consumed mainly by camels. Consequently, it gains a competitive advantage over more palatable species and may cause substantial economic losses to livestock production. Similar patterns have been reported in Uzbekistan, where the highest numbers of invasive species in natural habitats were also recorded in riparian and meadow ecosystems [19], supporting the generality of this pattern. Accordingly, we predict that zonal desert ecosystems will contain the lowest numbers of invasive species because arid conditions constrain the invasion process. However, this pattern is likely to apply primarily to undisturbed zonal communities, whereas intrazonal habitats, including oases, floodplains, and irrigated landscapes, may remain highly susceptible to biological invasions.

In the neighbouring Altai region, the proportion of alien species has been assessed across all major vegetation types except wetlands. Forest communities contained the lowest proportion of alien plants, accounting for only 1.7% of the total flora [80]. Comparable studies have not yet been conducted in the forest and forest-steppe communities of Kazakhstan, and, therefore, the proportion of alien species in these vegetation types remains unknown. Nevertheless, the absence of widespread reports of alien plants from forest communities suggests that the invasion process in the neighbouring forested regions of Kazakhstan is still at an early stage. At the same time, the occurrence of *Lupinus polyphyllus* L. in forest communities indicates that the establishment of individual invasive species has already begun and may precede their further spread. However, the extent of this process remains unclear and requires targeted floristic and phytosociological investigations.

Overall, the available evidence suggests that floodplain and meadow ecosystems currently represent the natural habitats most vulnerable to alien plant invasions in Kazakhstan, whereas zonal desert ecosystems remain comparatively weakly invaded. Nevertheless, increasing anthropogenic disturbance and ongoing climate change are likely to accelerate the establishment and naturalization of alien species in these ecosystems. This highlights the need for long-term monitoring, early detection of invasive species, and timely management measures to prevent their further spread.

The results further demonstrate that the majority of alien plant species in Kazakhstan remain associated with anthropogenically disturbed habitats. This pattern represents one of the defining characteristics of alien floras worldwide and reflects the central role of human activities in plant dispersal and establishment [12,14]. Disturbed habitats function as primary entry points and centres of establishment for alien species, whereas successful colonization of natural ecosystems occurs considerably less frequently. These findings emphasize the importance of transportation corridors, agricultural landscapes, urban environments, and other human-modified ecosystems as the principal pathways facilitating the spread of alien plants throughout Kazakhstan.

The preparation of the present checklist was accompanied by the compilation of a preliminary checklist of cultivated vascular plants of Kazakhstan [81], which enabled a more reliable distinction between cultivated species and genuinely naturalized alien plants. This approach minimized the inclusion of taxa lacking convincing evidence of escape from cultivation or persistent occurrence outside cultivation. Consequently, several species regarded as alien in neighbouring countries were excluded from the present checklist because no reliable herbarium specimens or recent field observations confirming their past or present occurrence in Kazakhstan were available. Likewise, several taxa previously reported from the country were excluded following a critical reassessment of the available taxonomic evidence [82,83]. This conservative, evidence-based approach substantially improves the reliability of the national checklist while minimizing the inclusion of erroneous records. Notably, the status of several taxa in our database remains uncertain. This especially refers to species known only from a few localities or isolated records, for which available evidence is still insufficient to confirm the existence of self-sustaining populations. Such uncertainty is common to most national checklists of alien floras and is gradually resolved as long-term monitoring data accumulate [4,47].

Particular attention should be given to the species exhibiting high invasive potential. Our assessment confirmed the occurrence of 20 invasive alien species in Kazakhstan, more than half of which belong to the Asteraceae family. A similar pattern has been reported from both Kyrgyzstan [17] and Uzbekistan [19], where representatives of Asteraceae also dominate the invasive component of the alien flora. This consistency suggests that the processes governing the formation of the most aggressive fraction of the alien flora are largely shared across Central Asia. At the same time, differences in the invasion status of individual species among neighbouring countries indicate that naturalization and invasion remain ongoing processes and that invasion assessments should be regularly updated as new evidence becomes available.

## 4. Materials and Methods

The distribution of vascular plants within Kazakhstan follows the floristic regionalization proposed by Pavlov [84], which divides the country into 29 floristic regions and 7 subregions (Figure 5).

As a taxonomic baseline, we used the national checklist of vascular plants of Kazakhstan compiled by Abdulina [35]. Accepted names of species and subspecies, genera, and families, together with their synonyms, were subsequently verified using Plants of the World Online [85].

Taxon-level distributional information within Kazakhstan was updated through a comprehensive review of regional floras, herbarium collections, our own field observations, and published taxonomic revisions and monographs.

Historical distribution data were extracted from Flora of Kazakhstan (nine volumes) [84,86,87,88,89,90,91,92,93] and Conspectus Florae Asiae Mediae (eleven volumes) [94,95,96,97,98,99,100,101,102,103,104]. We also examined numerous publications dealing with the regional floras of Kazakhstan and adjacent territories [27,30,32,48,53,54,105,106,107,108,109,110,111,112].

Particular attention was given to publications documenting new records, species distributions, naturalization, and invasion status of alien plants in Kazakhstan and neighbouring regions [18,20,21,22,23,24,33,53,113,114,115,116,117,118,119,120,121]. In addition, the Global Biodiversity Information Facility [122] and the community-science platforms iNaturalist and Plantarium [123,124] were examined for supplementary occurrence records. Community-science records were subjected to critical taxonomic screening before inclusion in the checklist. Photographs were examined for diagnostic morphological characters, and records with ambiguous identifications or insufficient photographic evidence were excluded. For taxonomically difficult taxa, community-science records were not accepted on the basis of platform identifications alone; where such records were considered, their identification was verified through consultation with specialists familiar with the respective taxonomic groups. Establishment status was assigned conservatively for taxa documented primarily by personal and community-science observations: in the absence of independent evidence of self-sustaining populations, such taxa were classified as casual rather than naturalized.

At the current stage, field observations were opportunistic rather than part of a standardized country-wide sampling programme and were used as supplementary evidence for taxon occurrence and distribution, rather than for estimating regional abundance, sampling effort, or population density. This approach enabled us to refine the known distributions of individual taxa and to incorporate recent records and documented range extensions within Kazakhstan in the current absence of dedicated surveys and occurrence databases.

The resulting taxonomically verified checklist and associated distributional data were organised into the Checklist of Alien Vascular Plants of Kazakhstan dataset and published through the Global Biodiversity Information Facility [125]. Distribution records correspond to the floristic regions and subregions of Kazakhstan following Pavlov [84], with geographic coordinates represented by the centroids of the respective floristic units. These coordinates therefore indicate regional occurrence rather than precise species localities.

The taxonomic arrangement and family circumscriptions followed PPG I [126] for lycophytes and ferns, Christenhusz et al. [127] for gymnosperms, and APG IV [128] for angiosperms. The authorship of taxon names was obtained from the International Plant Names Index [129] and verified using the protologues. For quantitative analyses, species, subspecies, and hybrid taxa were treated as equivalent taxonomic units. Nomenclatural changes and the most commonly used synonyms relative to the previous national checklist are provided in Table S1. Because the dataset includes taxa of different ranks, the term taxon (plural taxa) is used throughout the manuscript when referring collectively to entries in the checklist.

Following the methodology applied in the checklist of alien plants of Kyrgyzstan [17], we compiled a set of biological and ecological attributes for each alien taxon recorded in Kazakhstan. These attributes included life cycle, native geographical range, occurrence in natural ecosystems, weed status, association with particular crop types, cultivation history (ornamental, edible, technical, fodder, or medicinal), and occurrence in ruderal and roadside habitats.

Native-range assignments were based on recent taxonomic and floristic literature and authoritative databases, including Plants of the World Online, and were cross-checked against regional floras and taxonomic revisions. For taxa with broad or complex native distributions, we assigned the biogeographical region that best represented the principal native range recognized in the literature. Particular caution was applied to taxa whose native ranges approach the borders of Kazakhstan; in ambiguous cases, taxa were not treated as alien unless there was sufficient evidence of human-mediated introduction. Taxa of uncertain origin associated with a long history of cultivation were evaluated individually on the basis of available historical, floristic, and taxonomic evidence.

In this study, natural ecosystems were defined as habitats in which vegetation composition and structure are predominantly maintained by natural ecological processes and are not directly created or regularly maintained by human activity. Human-modified habitats included agricultural land, settlements, roadsides, railway corridors, ruderal sites, plantations, and other habitats whose structure or disturbance regime is primarily shaped by human activities. Weed status was treated as an agronomic attribute independent of establishment and invasive status. Accordingly, an alien taxon could be classified as a weed regardless of whether it was casual, naturalized, or invasive.

Establishment status was assessed following Richardson et al. [12] and Blackburn et al. [130]. Alien taxa were defined as taxa occurring outside their native range as a result of intentional or unintentional human-mediated introduction and were classified as casual, naturalized, or unresolved. Casual taxa were those that occurred outside cultivation but did not form self-sustaining populations, whereas naturalized taxa were those for which stable, self-reproducing populations had been documented. Taxa for which the available evidence was insufficient to distinguish between casual and naturalized status were classified as unresolved. A taxon was considered naturalized when evidence of a self-sustaining population was available from at least one locality in Kazakhstan, irrespective of the extent of its distribution within the country.

Invasive status was evaluated separately from establishment status. A naturalized taxon was regarded as invasive when there was evidence of sustained spread beyond the sites of introduction. This classification was based on evidence of spread and did not, by itself, imply a particular level of environmental impact. Environmental impacts were assessed independently using the EICAT protocol [37]. EICAT categories were assigned by the authors specifically for Kazakhstan on the basis of published evidence of ecological impacts; distributional records were used as supporting context rather than as evidence of impact.

Because much of the information on establishment, spread, and impact was derived from published sources and available distribution records, these assessments should be regarded as a national-scale synthesis based on the best available evidence rather than as a substitute for targeted field studies. Field investigations remain necessary to evaluate abundance, population dynamics, rates of spread, and local ecological effects, and some classifications may require revision as new evidence becomes available.

Taxa native to any part of Kazakhstan were excluded from the checklist, even when they may occur outside their native regional range elsewhere within the country. For taxa introduced through cultivation, cultivation history was recorded separately from evidence of escape. Such taxa were included as alien only when casual occurrences outside cultivation or established naturalized populations were documented.

Taxa were additionally classified according to their residence time in Kazakhstan as archaeophytes or neophytes following Sennikov and Lazkov [17]. Archaeophytes were defined as taxa introduced before AD 1800, prior to the beginning of systematic botanical exploration in the region, whereas taxa introduced from AD 1800 onwards were classified as neophytes. Analyses of naturalization success were descriptive; no inferential statistical tests were performed because the underlying data were heterogeneous in source, space, and time and were derived from unequal sampling effort. Selected alien taxa recorded in Kazakhstan are illustrated in Figure 6. Taxon-specific sources supporting occurrence records and identifications are provided in Table S1 [131,132,133,134,135,136,137,138,139,140,141,142,143,144,145,146,147,148,149,150,151,152,153,154,155,156,157,158,159,160,161,162,163,164,165,166,167,168,169,170,171,172].

## 5. Conclusions

This study provides the first comprehensive assessment of the alien vascular flora of Kazakhstan and substantially advances current knowledge of its taxonomic composition, geographic origins, spatial distribution, and degree of naturalization. However, further investigations should focus particularly on poorly explored regions, including the desert landscapes of western Kazakhstan, the Aral Sea region, the Ustyurt Plateau, and several mountain systems, where dedicated studies of alien plants have thus far been limited. Comparisons with neighbouring countries demonstrate that, although the alien flora of Kazakhstan shares several general characteristics with other Central Asian alien floras, including the predominance of Mediterranean-origin taxa and annual species, it also exhibits distinctive features associated with the country’s vast territory, pronounced environmental heterogeneity, and unique history of plant introductions. The marked variation in recorded alien taxon richness among floristic regions probably reflects a combination of environmental heterogeneity, land-use and introduction history, and uneven floristic recording effort. At the same time, our analysis revealed considerable geographical variation in floristic knowledge and significant gaps in the current understanding of alien plant distributions across Kazakhstan. These findings emphasize the need for continued floristic surveys, long-term monitoring, and periodic revision of the national alien plant checklist to facilitate the timely detection of newly introduced species, improve the assessment of naturalization processes, and refine invasion risk evaluations. Future integration of biotechnological, molecular-genetic (eDNA metabarcoding, chromosomal-scale genome assembly), metabolomic (mass spectrometry imaging), ecological niche modelling and remote sensing approaches will be essential for enhancing the early detection, monitoring and risk assessment of alien species in Kazakhstan.

## Figures and Tables

**Figure 1:**
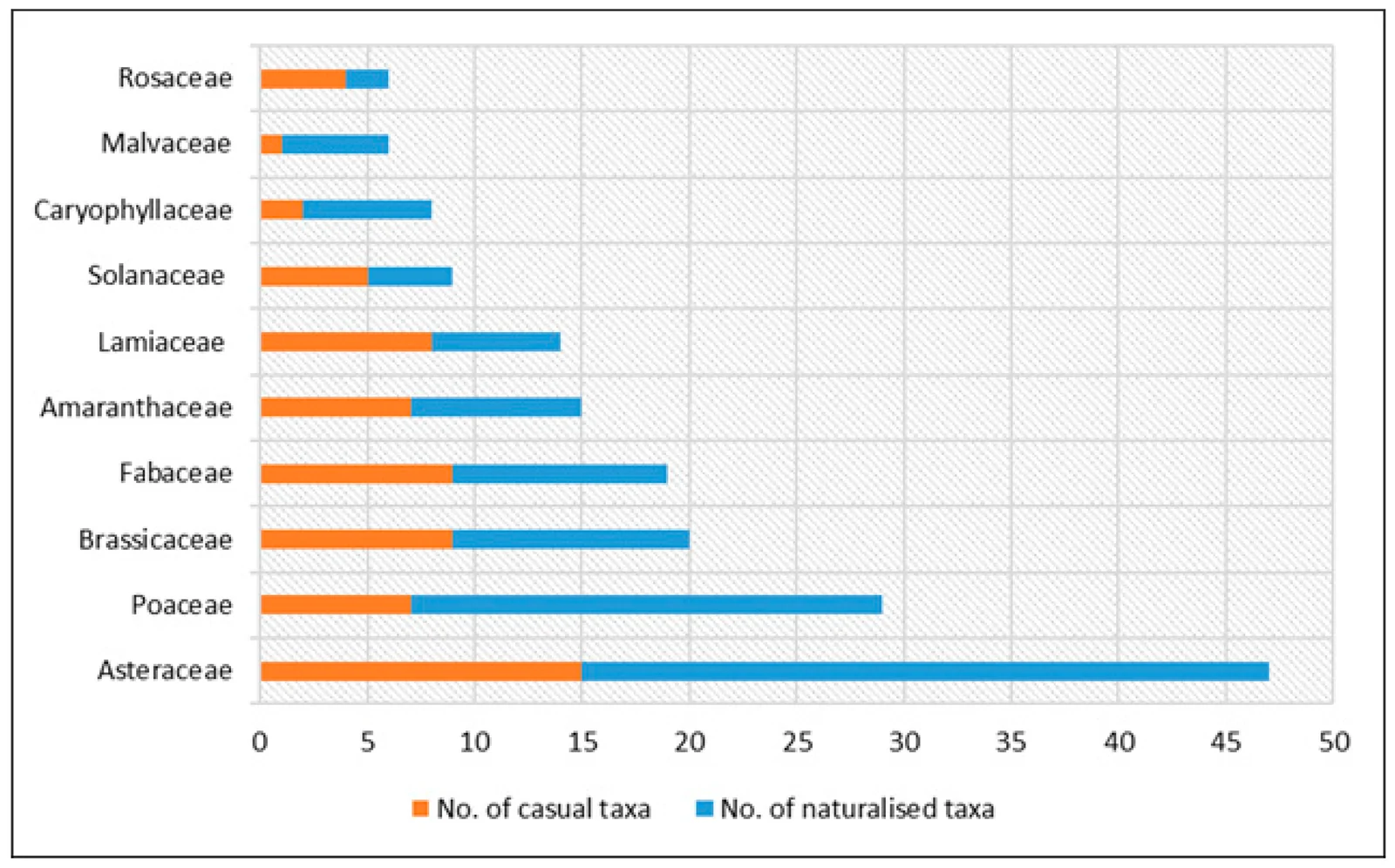
Most represented families in the alien flora of Kazakhstan. Families represented by at least six alien taxa are shown; bars indicate establishment status. Taxa of unresolved establishment status are not included in the bars.

**Figure 2:**
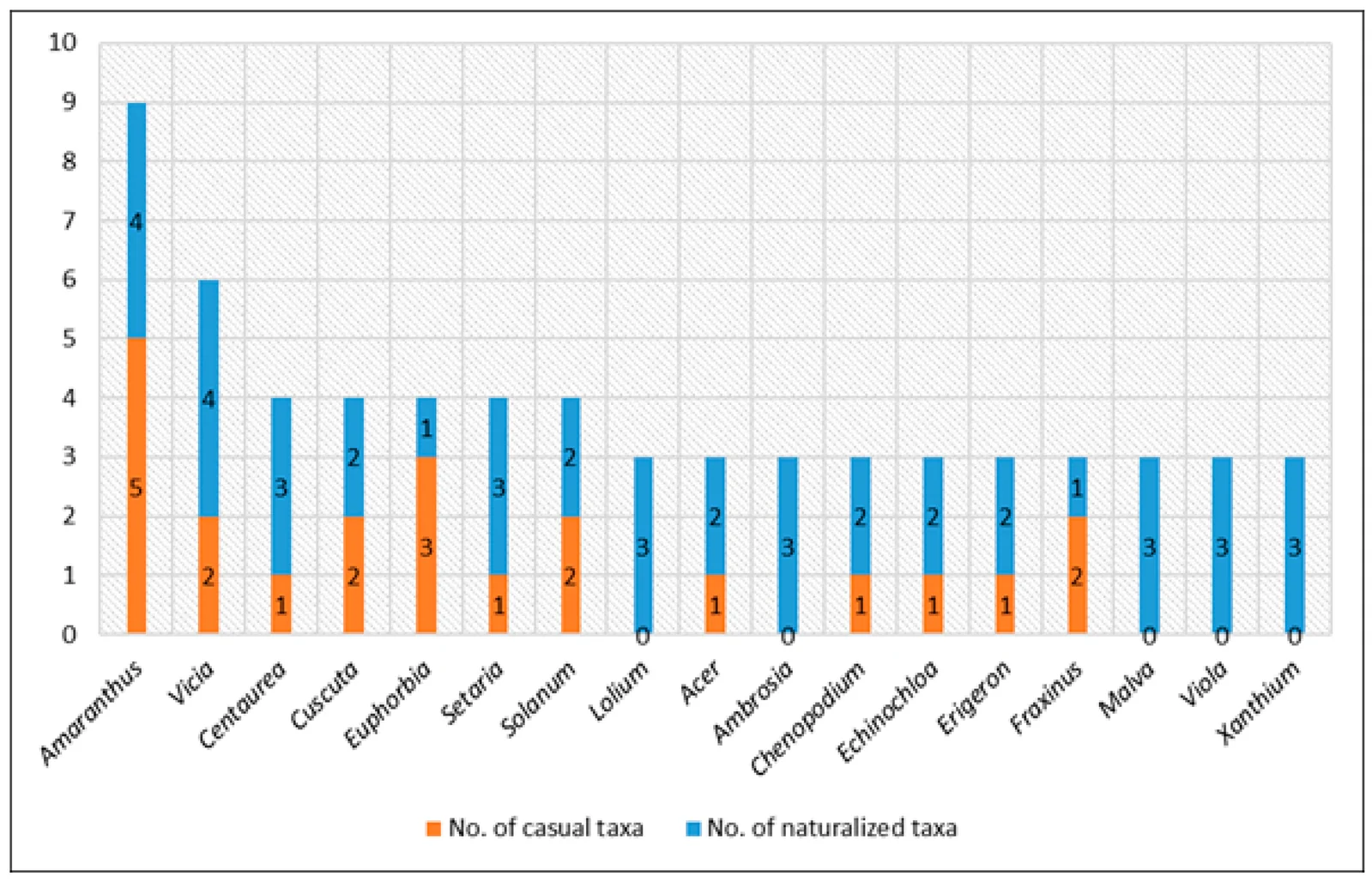
Most taxon-rich genera of the alien flora of Kazakhstan. Genera represented by at least three alien taxa are shown; bars indicate establishment status. Taxa of unresolved establishment status are not included in the bars.

**Figure 3:**
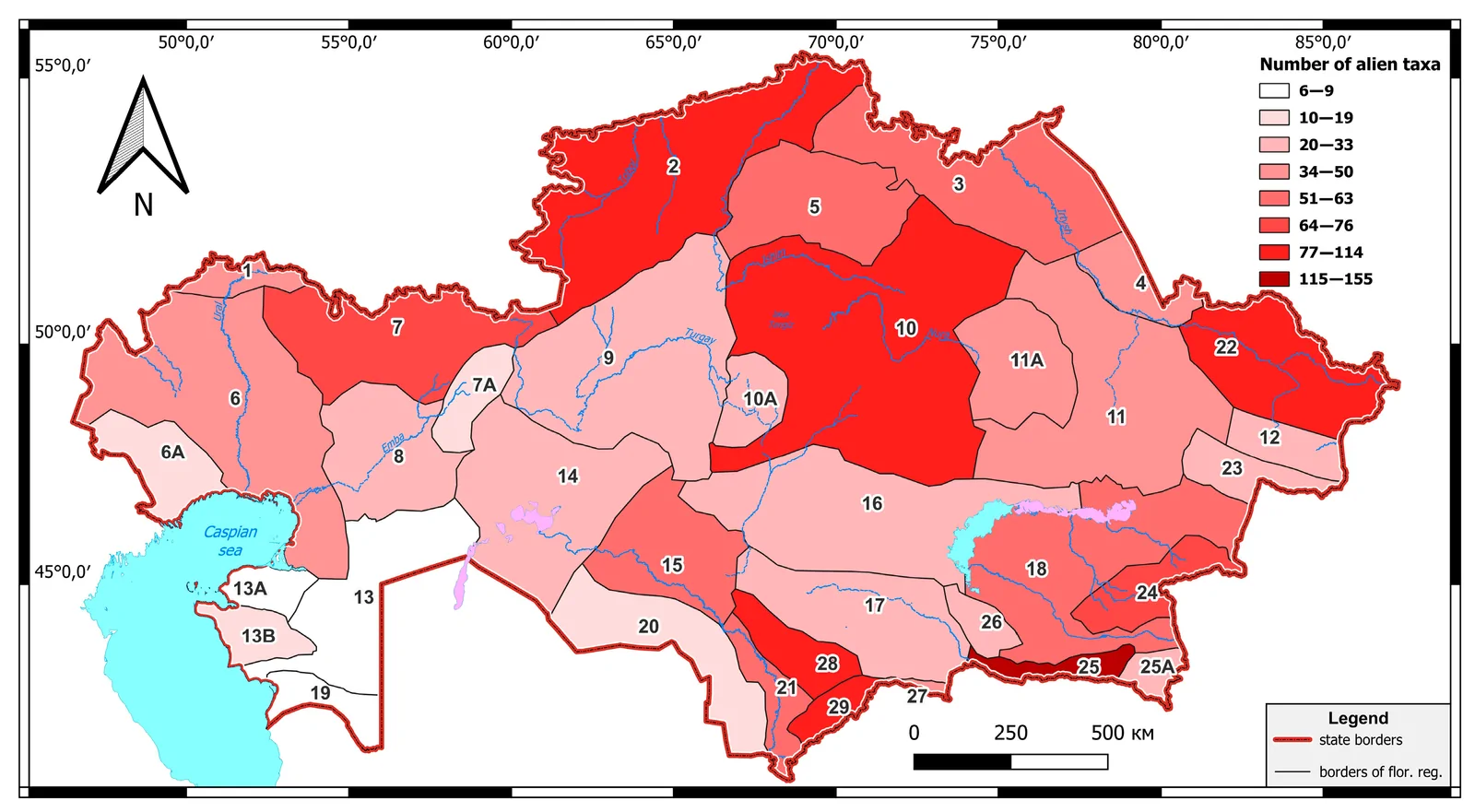
Spatial variation in the number of alien vascular plant taxa across the floristic regions of Kazakhstan. Colour intensity indicates the number of alien taxa recorded in each region. For codes of floristic regions, see Section 4.

**Figure 4:**
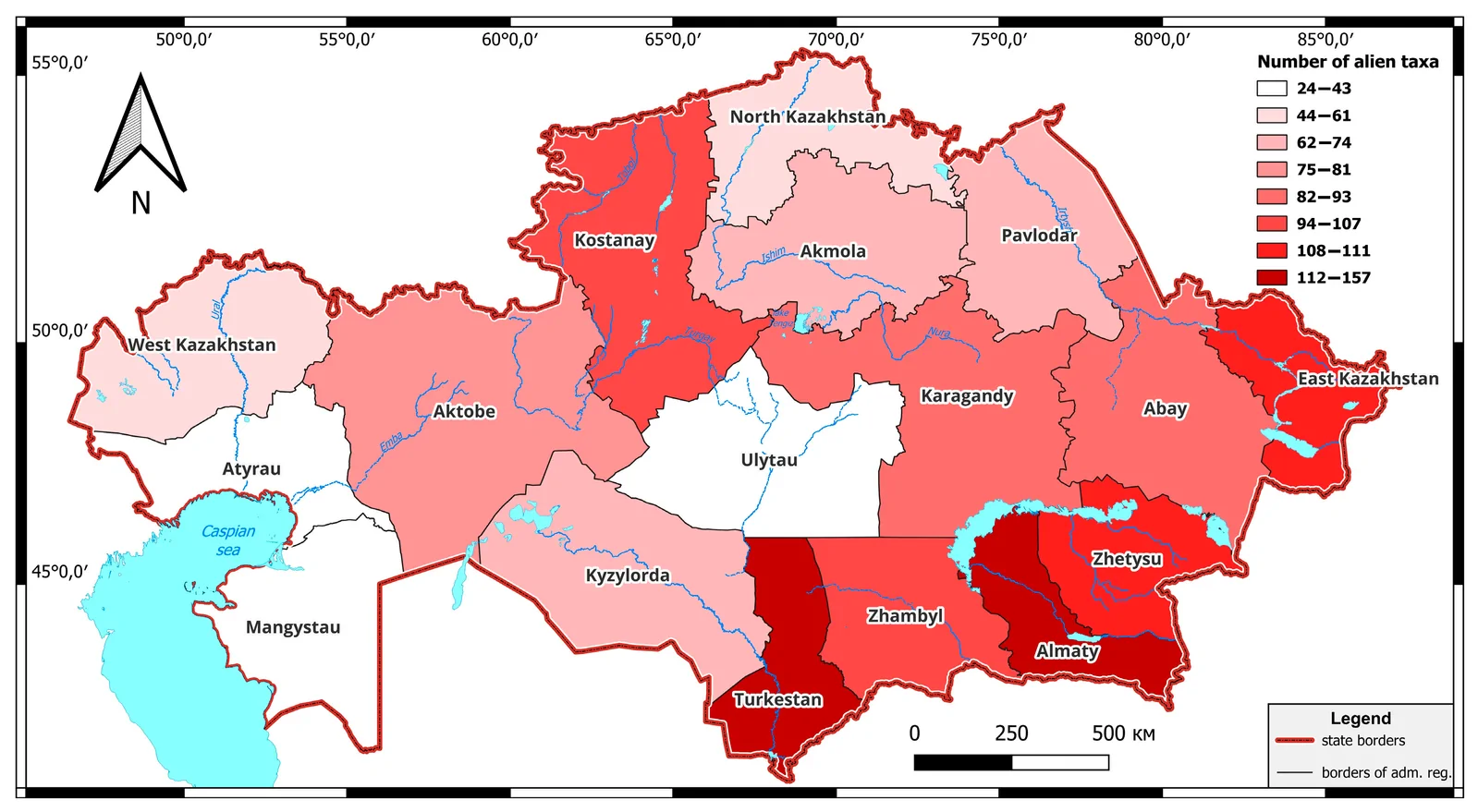
Distribution and richness of alien vascular plant taxa across the administrative regions of Kazakhstan. Colour intensity indicates the number of alien taxa recorded in each region.

**Figure 5:**
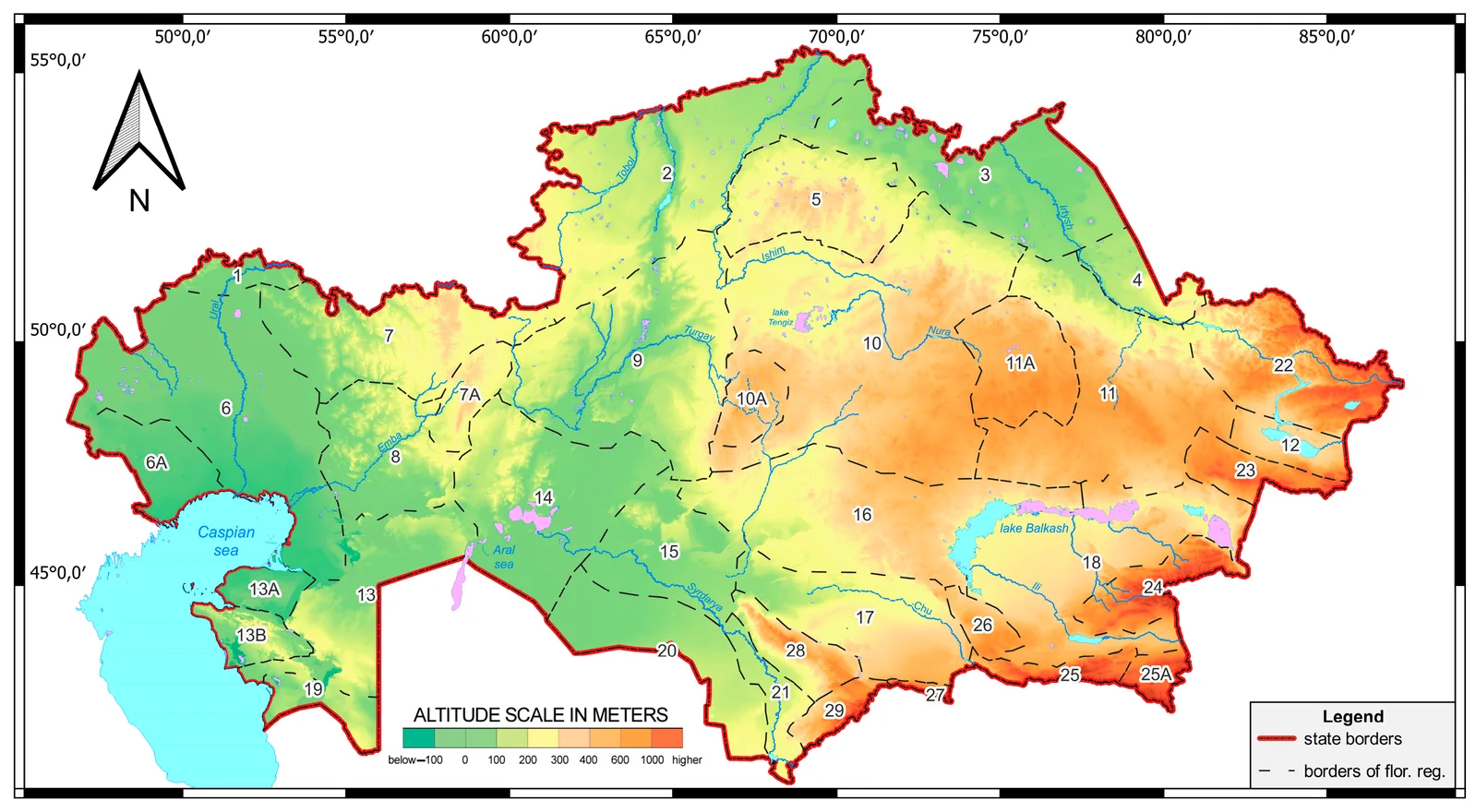
The floristic regions and topography of Kazakhstan: 1—Syrt, 2—Tobol-Ishim, 3—Irtysh, 4—Semipalatinsk pine forest, 5—Kokchetav, 6—Caspian, 6A—Bukeev, 7—Aktobe, 7A—Mugojary, 8—Emba, 9—Turgay, 10—Western Upland, 10A—Ulutau, 11—Eastern Upland, 11A—Karkaraly, 12—Zaysan, 13—Northern Ustyrt, 13A—Buzachi, 13B—Mangyshlak, 14—Aral, 15—Kyzylorda, 16—Betpak-Dala, 17—Moiynkum, 18—Balkhash Alakol, 19—Southern Ustyrt, 20—Kyzylkum, 21—Turkestan, 22—Altai, 23—Tarbagatai, 24—Dzungarian Alatau, 25—Trans-Ili Kungey Alatau, 25A—Ketmen-Terskey Alatau, 26—Chu-Ili Range, 27—Kyrgyz Alatau, 28—Karatau, 29—Western Tian-Shan.

**Figure 6:**
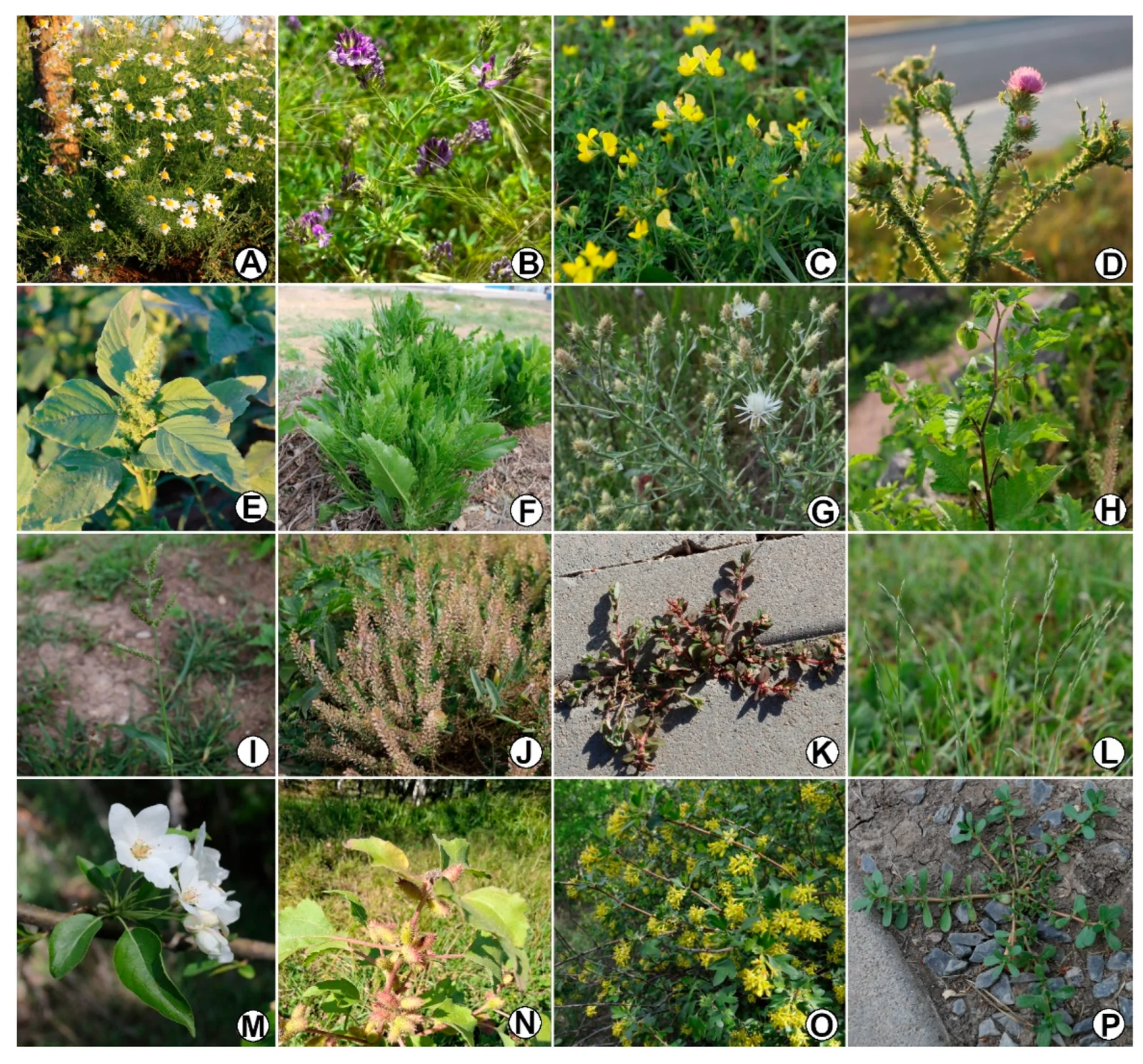
Representative alien vascular plant species recorded in Kazakhstan: (**A**) *Tripleurospermum inodorum* (L.) Sch.Bip., (**B**) *Medicago sativa* L., (**C**) *Lotus corniculatus* L., (**D**) *Carduus acanthoides* L., (**E**) *Amaranthus retroflexus* L., (**F**) *Armoracia rusticana* G. Gaertn., B. Mey. & Scherb., (**G**) *Centaurea diffusa* Lam., (**H**) *Nicandra physalodes* (L.) Gaertn., (**I**) *Echinochloa crus-galli* (L.) P. Beauv., (**J**) *Lepidium densiflorum* Schrad., (**K**) *Euphorbia maculata* L., (**L**) *Lolium perenne* L., (**M**) *Malus baccata* (L.) Borkh., (**N**) *Xanthium orientale* L., (**O**) *Ribes aureum* Pursh, (**P**) *Portulaca oleracea* L. (Photographed by D. Alibekov).

**Table 1:** Geographic origin and naturalization status of alien vascular plants in Kazakhstan.

Native Range	Total	Casual	Naturalized	Unresolved
Mediterranean+	105	30	74	1
America	60	34	26	0
Europe	34	20	14	0
Asia	29	15	13	1
Asia Minor–Caucasus–Iran	12	7	5	0
Cultivation	11	7	4	0
Africa–Asia	9	6	3	0
Africa	1	0	1	0
Eurasia	1	0	1	0
Asia–Australasia	1	1	0	0
Total	263	120	141	2

Note: Mediterranean+ includes taxa with native ranges centred in the Mediterranean region but extending into adjacent geographical areas.

**Table 2:** Documented cultivation uses and naturalization status of alien plant taxa in Kazakhstan.

Documented Cultivation Use	Total	%	Casual	Naturalized	Unresolved
Ornamental	57	21.67	34	23	0
Edible	24	9.13	18	6	0
Fodder	19	7.22	7	12	0
Medicinal	9	3.42	5	4	0
Technical	7	2.66	3	4	0
No documented cultivation	150	57.03	55	93	2
Total	263	100.00	120	141	2

Note: Cultivation-use categories are not mutually exclusive; three taxa are assigned to more than one use category. Therefore, category totals and percentages should not be summed. The row ‘No documented cultivation’ is mutually exclusive with all cultivation-use categories.

**Table 3:** Invasive alien vascular plant taxa of Kazakhstan and their Environmental Impact Classification for Alien Taxa (EICAT) impact categories.

Scientific Name	Family	Life Cycle	Residence Status	Native Range	Distribution in Kazakhstan	EICAT Impact Category
*Centaurea diffusa*	Asteraceae	Biennial	Neophyte	Mediterranean	Regional	Major (MR)
*Cuscuta campestris*	Convolvulaceae	Annual	Neophyte	America	Regional	Major (MR)
*Cyclachaena xanthiifolia*	Asteraceae	Annual	Neophyte	America	Regional	Major (MR)
*Acer negundo*	Sapindaceae	Perennial	Neophyte	America	Widespread	Moderate (MO)
*Ailanthus altissima*	Simaroubaceae	Perennial	Neophyte	China	Local	Moderate (MO)
*Ambrosia artemisiifolia*	Asteraceae	Annual	Neophyte	America	Regional	Moderate (MO)
*Ambrosia psilostachya*	Asteraceae	Perennial	Neophyte	America	Local	Moderate (MO)
*Ambrosia trifida*	Asteraceae	Annual	Neophyte	America	Local	Moderate (MO)
*Cuscuta chinensis*	Convolvulaceae	Annual	Neophyte	China	Widespread	Moderate (MO)
*Elodea canadensis*	Hydrocharitaceae	Perennial	Neophyte	America	Widespread	Moderate (MO)
*Erigeron annuus*	Asteraceae	Annual	Neophyte	America	Local	Moderate (MO)
*Erigeron canadensis*	Asteraceae	Annual	Neophyte	America	Regional	Moderate (MO)
*Euonymus europaeus*	Celastraceae	Perennial	Neophyte	Europe	Local	Moderate (MO)
*Fraxinus excelsior*	Oleaceae	Perennial	Neophyte	Europe	Local	Moderate (MO)
*Hordeum jubatum*	Poaceae	Perennial	Neophyte	America	Regional	Moderate (MO)
*Phelipanche aegyptiaca* (Pers.) Pomel	Orobanchaceae	Annual	Archaeophyte	Mediterranean	Local	Moderate (MO)
*Pilosella aurantiaca*	Asteraceae	Perennial	Neophyte	Central Europe	Local	Moderate (MO)
*Senecio viscosus*	Asteraceae	Annual	Neophyte	Central Europe	Local	Moderate (MO)
*Xanthium orientale*	Asteraceae	Annual	Neophyte	America	Regional	Moderate (MO)
*Xanthium spinosum*	Asteraceae	Annual	Neophyte	America	Regional	Moderate (MO)

## Data Availability

The data underlying this study are available in the Supplementary Materials (Table S1) and through the Global Biodiversity Information Facility (GBIF) as the Checklist of Alien Vascular Plants of Kazakhstan dataset (Version 1.6), available online: https://doi.org/10.15468/tz64jk (accessed on 3 September 2026).

## Supplementary Materials

The following supporting information can be downloaded at https://www.mdpi.com/article/10.3390/plants15192934/s1: Table S1, Checklist of alien vascular plants of Kazakhstan (xlsx).
